# The Management of Knee Osteoarthritis in the Real World: An Italian National Survey

**DOI:** 10.3390/jcm13133704

**Published:** 2024-06-25

**Authors:** Sefora Codazza, Paola Emilia Ferrara, Mariantonietta Ariani, Giorgio Ferriero, Gianpaolo Ronconi

**Affiliations:** 1 University Polyclinic Foundation A. Gemelli IRCCS, 00168 Rome, Italy; sefora.codazza@guest.policlinicogemelli.it (S.C.); paolaemilia.ferrara@policlinicogemelli.it (P.E.F.); 2 Department of Neurosciences, Sense Organs and Thorax, Catholic University of the Sacred Heart, 00168 Rome, Italy; 3 Unit of Physical and Rehabilitation Medicine, Istituti Clinici Scientifici Maugeri IRCCS, 21049 Tradate, Italy; giorgio.ferriero@icsmaugeri.it; 4 Department of Biotechnology and Life Sciences, University of Insubria, 21110 Varese, Italy; 5 Department of Rehabilitation, Catholic University of the Sacred Heart, 00168 Rome, Italy; gianpaolo.ronconi@unicatt.it

**Keywords:** knee osteoarthritis, rehabilitation, physical therapy, pharmacological therapy

## Abstract

**Background:** Knee osteoarthritis is a degenerative and inflammatory disease causing pain and worsening patients’ quality of life. Various conservative treatment options exist, but a gap between scientific evidence and clinical practice is still present. The aim of this prospective multicenter observational study is to describe the real outpatient territorial management of patients with knee osteoarthritis and to analyze the correlation between the anthropometric and clinical characteristics of the population of patients suffering from symptomatic knee osteoarthritis who were screened in the national survey. **Methods:** The educational national project was divided into three modules: the first and the last through webinars; and the second held in daily practice. The participants had to register structured observations, which were then stored in a national database and analyzed in order to identify correlations. The subgroups were stratified by body composition, radiological severity of knee osteoarthritis, pain, and functional ability. **Results:** The project has been joined by 155 physicians, and 2.656 observations about real-world outpatients being treated for knee osteoarthritis in Italy were collected. Data relating to real-world pharmacological and rehabilitation therapies in correlation with body composition, the radiological severity of knee osteoarthritis, pain, and functional ability were reported. **Conclusions:** Currently, there are no standardized protocols using effective combinations of therapeutic exercises, physical agents, and medications to control the progression of knee osteoarthritis. This real-word national survey proved to be useful for describing the current state of the art of therapeutic management of knee osteoarthritis and for emphasizing the need to fill the gap between scientific evidence and clinical practice.

## 1. Introduction

Knee osteoarthritis (KOA) is the most common degenerative joint disease in the general population [1]. KOA is typically characterized by lesions in the articular or subchondral bones, ligaments, synovium, joint capsule, and periarticular muscular structures [2]. KOA is associated with population obesity [3] and aging, with nearly 30% of individuals older than 45 years presenting radiographic signs of knee osteoarthritis, about half of whom have knee symptoms [4]. KOA causes a significant reduction in individual autonomy and a deterioration in the patient’s quality of life and increases the risk of all-cause mortality [5] and public health costs [6]. Therefore, the identification of effective therapies for the containment of symptoms and disability is of great interest to the international scientific community.

The therapeutic management of KOA can be divided into conservative therapies, which comprise education, exercise, weight reduction, orthosis, and drugs, or surgical treatment, which is performed when non-surgical strategies fail. Surgical procedures are a last resort for end-stage KOA, performed by the Orthopedic Surgeons [7].

Of the non-surgical treatments, pharmacological therapy is often the first strategy prescribed by general practitioners and involves the use of paracetamol, topical or oral non-steroidal anti-inflammatory drugs (NSAIDs), or intra-articular corticosteroids [8]. In addition, rehabilitation is usually performed as a second step when oral pharmacological treatments do not control the acute symptoms of KOA, and when secondary pain syndromes occur in other joint sites due to the walking deficit that occurs due to the impaired knee function becoming chronic. However, patients often receive confused messages about whether or not to undertake therapeutic exercise: they are often told that joint rest is harmful, but in some cases, people who have followed an exercise program report that they have experienced an exacerbation of their symptoms and are reluctant to continue; in other cases, they are alerted that activity ‘wears out’ joints, and they may then experience that rest exacerbates joint stiffness and pain [9].

Although a variety of evidence-based guidelines for the clinical management of knee osteoarthritis have been developed by national and international organizations [10,11] due to the improved understanding of the underlying pathogenetic mechanisms of KOA, in daily clinical practice, therapeutic interventions for KOA are often based on individual practitioners’ experience and not on standardized recommendations.

The need for practical therapeutic stepwise guidance for KOA is demonstrated by the results of our previous real-world analysis of the real outpatient management of patients with KOA in Italy according to the European Society for Clinical and Economic Aspects of Osteoporosis, Osteoarthritis and Musculoskeletal Diseases (ESCEO) guidelines [7]. The real-world national survey showed that the rising use of anti-inflammatory drugs and analgesics is often not supported by prescriptive appropriateness, and the increased utilization of joint nutraceuticals and viscosupplementation is not guided by an updated algorithm of available therapeutic molecules.

Our previous real-world data [7] demonstrated a predominant use of oral NSAIDs as the first-line treatment, when they are strongly recommended only in the topical form if the patient is still symptomatic after establishing appropriate background therapy with Symptomatic Slow-Acting Drugs for Osteoarthritis (SYSADOAs) and when rescue analgesic therapy with paracetamol has failed. In addition, selective or non-selective oral NSAIDs, according to ESCEO experts, should be considered advanced pharmacological treatment, to be administered in intermittent or prolonged cycles in patients with persistent symptoms based on a risk profile [11].

Regarding paracetamol, our previous real-world observations [7] have shown that it is commonly used as a background therapy, although to a lesser extent than NSAIDs. However, paracetamol is indicated as a short-term rescue analgesic therapy at doses no greater than 3 g/day, and not as a background drug, because of its minimal efficacy and the increasing evidence of multisystemic adverse events [12].

The symptomatic SYSADOAs are widespread compounds that are commonly used in clinical practice as a secondary pharmacological tool and only as a support of primary pharmacological therapy with NSAIDs, as shown in our previous analysis of real-world data [7]. SYSADOAs, and in particular crystal-line glucosamine sulfate or chondroitin sulfate, are the strongly recommended background approach for symptomatic knee osteoarthritis [11]. 

The aim of this prospective multicenter observational study is to describe the correlation between anthropometric and clinical characteristics, such as pain and functional limitation, of the population of patients suffering from symptomatic KOA screened in the national ESCEO survey [7].

Furthermore, we will report data relating to real-word pharmacological treatments, rehabilitation therapy and intra-articular injections for the population stratified by body composition, radiological severity of KOA, and functional ability. 

## 2. Methods 

**Figure 1:**
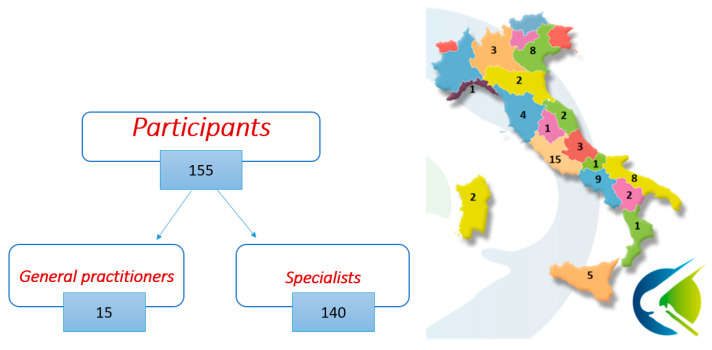
Participants of the educational project “Clinical practice evidence for knee osteoarthritis” in the Italian regions.

The educational project “Clinical practice evidence for knee osteoarthritis” was developed from the 15 October 2020 to 17 May 2021 and was designed as a national medical training course in a blended modality. The course has been joined by a total of 155 Italian physicians, consisting of 15 general practitioners and 140 specialists with different clinical and surgical specializations (Physical and Rehabilitation Medicine, Rheumatology, Orthopedics, Geriatrics, Sports Medicine, Anesthesiology), as showed in Figure 1. 

The main goals of the educational project were as follows: to provide basic knowledge on the clinical management of KOA in the medium- and long term, to assess the real occurrence of knee pain in Italian outpatient settings, to evaluate the efficacy and safety of the drugs currently available, and to detect prevention and treatment models. Finally, the evidence that emerged from clinical practice was compared with the ESCEO guidelines for KOA [11] to better understand the gap between daily clinical practice and evidence-based medicine.

The training course was structured into three different modules:

- A first session of Remote Training via a webinar, during which the evidence on the pathophysiology of KOA and the current therapeutic strategies were presented. In this section, it was explained how to use the Activity Book, which is a data collection sheet consisting of 10 questions where clinical features and prescribed therapies are recorded by the physicians in their real clinical practice for each patient affected by KOA. The Activity Book represented the real-world collection tool for the national survey. Each participant was given a maximum of 40 Activity Books, corresponding to 40 patients with KOA.
- A daily practice session, which consisted in the completion by each participant of the Activity Books during the clinical and physical examination of the patients with KOA. The doctors had an Activity Book available for each patient enrolled, where they had to collect different types of data about KOA. First of all, the demographic and anamnestic data and the anthropometric parameters were collected; then, the presence of local signs (edema, thermotact, range of motion) and symptoms (pain, stiffness) of the affected knee was assessed, and also, the knee’s range of motion was measured using a goniometer. Then, in the Activity Book, the physicians were also asked to define the radiological severity of KOA and to check the functional characteristics and the motor abilities in the performance of activities of daily living for each patient with KOA using a dedicated locomotor scale. In the end, in the treatment section of the questionnaire, the physicians had to register all pharmacological therapies (oral and intra-articular) and rehabilitation carried out by the patient up to that point; the participants had to note down the new therapeutic prescription resulting from the structured evaluation according to the standardized questionnaire of the Activity Book for patients with symptomatic KOA.
- A second session of Remote Training through a webinar, in which the overall results were discussed by tutors, including the comparison of the real-world data collected from the practice session with the ESCEO guidelines [11].

The Activity Books were then collected from each participant, and the clinical and functional observations were extracted and stored in a national database. After this, the following groups of information were identified and tallied for the statistical analysis:

▪ The demographic and anthropometric characteristics of the examined population: age, weight, height, and Body Mass Index (BMI);
▪ The radiological severity of knee osteoarthritis according to the Kellgren–Lawrence (K-L) classification [13];
▪ The pain level, measured using the Visual Analogic Scale (VAS);
▪ The locomotor skills in walking and the functional abilities of daily living, assessed through the Geriatric Locomotive Function Scale (GLFS);
▪ The pharmacological therapy, the rehabilitation treatments, and the intra-articular knee injections before and after the clinical observation.

The sample was subsequently stratified by BMI into three groups (normal weight 18.5–24.9 kg/m^2^, overweight 25–29.9 kg/m^2^, and obese 30–60 kg/m^2^), by the radiological severity of knee osteoarthritis according to K-L classification into two groups (stage II and stage III), and finally, into two groups based on the usage or not of rehabilitative treatments. 

In addition, data regarding the oral and intra-articular pharmacological therapy before and after the clinical observation were extracted and analyzed in relation to the stratification by BMI, by K-L, and by whether a rehabilitation program was performed.

Statistical analysis was performed using SPSS 14.0 software (SPSS Inc., Chicago, IL, USA). Descriptive analysis was performed using standard procedures for the calculation of frequencies to measure the average position (arithmetic mean) and dispersion indicators. The eventual comparison between groups was carried out using Student’s *t* test with a *p*-level significance < 0.05.

## 3. Results 

A total of 2656 clinical observations from patients suffering from KOA were collected during the real-world educational project. The database analysis of the received information showed a global heterogeneity with a different frequency for each group; the missing data can be traced back to the items that were not filled in by each participant in the individual activity books, some of which were therefore incomplete and did not report all the information required by the specific sections. 

The demographic characteristics of the examined population are summarized in Table 1.

Table 2 reports the VAS and GLFS scores in relation to the sample stratifications for BMI, K-L grade, and rehabilitation therapy. 

Comparing pain and function in the three groups of patients stratified by their BMI, the analysis revealed a mean VAS score of 5.9 and a mean GLFS score of 7.1 for normal-weight patients (BMI 18.5–24.9), a mean VAS score of 6.5 and a mean GLFS score of 8.2 for overweight patients (BMI 25–29.9), and a mean VAS score of 6.3 and a mean GLFS score of 10.2 for obese patients (BMI 30–60).

Comparing pain, function, and BMI in the two groups of patients stratified by their K-L grade, the analysis revealed a mean VAS score of 6.2, a mean GLFS score of 7.6, and a mean BMI of 26.7 for the K-L II group and a mean VAS score of 6.4, a mean GLFS score of 11.3, and a mean BMI of 28.7 for the K-L III group.

The data analysis of pain and function in the two groups of patients divided by rehabilitation therapy showed a mean VAS of 6.1 and a mean GLFS of 8.8 in the group performing rehabilitation and a mean VAS of 6.2 and a mean GLFS of 7.6 in the group not performing rehabilitation.

**Figure 2:**
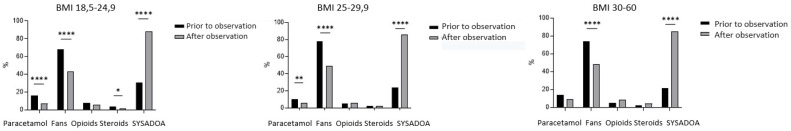
Pharmacological therapy prior to and after the clinical observation based on BMI. *, ****, ** significant differences between groups, *p* level <0.05 (CHI-square test with Yates corrections).

**Figure 3:**
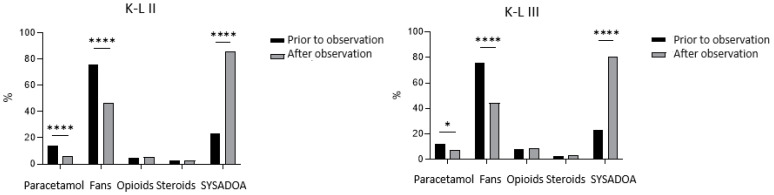
Pharmacological therapy prior to and after the clinical observation based on K-L. *, **** significant differences between groups, *p* level <0.05 (CHI-square test with Yates corrections).

**Figure 4:**
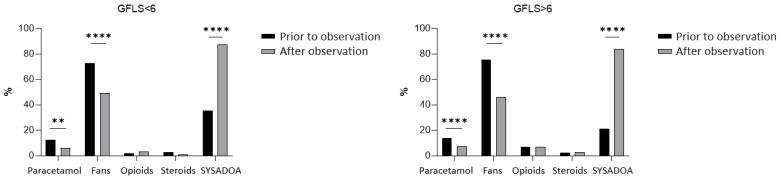
Pharmacological therapy prior to and after the clinical observation based on GLFS. ****, ** significant differences between groups, *p* level < 0.05 (CHI-square test with Yates corrections).

In Figure 2, Figure 3 and Figure 4, the drugs taken by patients before observation and after medical prescription are represented in relation, respectively, to the BMI (Figure 2), the K-L grade (Figure 3), and the GLFS (Figure 4).

The graphs of Figure 2 show a similar trend in drug use in the three groups classified by BMI. It is observed that obese patients showed a significant reduction in NSAIDs and increase in SYSADOA after the observation. The intake of paracetamol was reduced in the three groups. 

From the graphs of Figure 3, we can observe a similar trend in the use of drugs in the two groups based on K-L grade. It is observed that patients with more severe knee osteoarthritis (K-L III) present a significant reduction in NSAIDs and a significant increase in SYSADOA after the observation and a less significant reduction in paracetamol usage.

From the graphs in Figure 4, we can observe a similar trend in the use of drugs in the two groups organized based on GLFS. In both functional groups, a significant reduction in paracetamol and NSAID utilization and a significant increase in SYSADOAs therapy after observation are registered.

The percentage of intra-articular knee injections performed before and after observation in relation to the parameters of BMI, K-L, and GLFS and rehabilitation therapy are reported in Table 3.

An overall increase in the percentage of intra-articular injections performed after the observation was recorded in all patient groups stratified by BMI, K-L, and GLFS.

In the BMI groups, the following percentages were observed: 0.3% before observation and 6.8% after observation for normal-weight patients (n: 891); 0.6% before observation and 6.6% after observation for overweight patients (n: 1135); and 0.5% before observation and 6.4% after observation for obese patients (n: 409).

In the K-L groups, the following percentages were observed: 0.2% before observation and 8.6% after observation for K-L II group (n: 882) and 0.5% before observation and 8.7% after observation for K-L III group (n: 632).

Regarding the GLFS subclassification, values of 0% before observation and of 6.4% after observation were observed for the GLFS < 6 group (n: 828) and values of 0.5% before observation and of 7.9% after observation were observed for the GLFS > 6 group (n: 1797). Percentages of 0.5% before observation and 8.9% after observation were observed in patients who performed rehabilitation therapy (n: 1389) and a percentage of 0.3% before observation and 5.6% after observation were observed in patients who did not perform rehabilitation therapy (n: 1252).

## 4. Discussion

The aim of this prospective multicenter observational study is to illustrate the correlations between body composition and the radiological severity of osteoarthritis according to the Kellgren–Lawrence classification with respect to pain levels, measured with VAS, and locomotor abilities in walking and functional abilities in daily living, evaluated through GLFS, in a sample of patients suffering from symptomatic KOA in a real-life outpatient setting. Further analysis was then conducted by relating the aforementioned clinical and functional data to pharmacological therapy, rehabilitative treatments, and intra-articular knee injections before and after clinical observation was carried out by the participants in the educational project.

In our previous study [7], we described the real outpatient management of patients with KOA and compared real-life protocols with the ESCEO guidelines, which are evidence-based guidelines for the management ok KOA to relieve symptoms and slow cartilage degeneration, with a consequent improvement in quality of life and healthcare cost reduction.

The six-month research project involved 155 Italian general practitioners and specialists (Figure 1), who collected a total of 2656 clinical observations from patients suffering from knee osteoarthritis. The data collection during the practical session, with the compilation of Activity Books, enabled the evaluation of different treatments for knee osteoarthritis before and after the medical training course, particularly after the explanation of the ESCEO recommendations.

Regarding demographic data, the mean age of the observed population is 67.2 years, and thus in line with the currently known epidemiology for knee osteoarthritis, which is highly prevalent among people aged over 50 years, affecting more than 250 million people worldwide [14].

Regarding anthropometric characteristics, the majority of the sample was found to be overweight, with a mean value of BMI of 26.4, with homogeneous distribution between genders. Our data reflect the well-known evidence that obesity is a cardinal risk factor for the development of knee osteoarthritis, especially the finding described by Misra et al. [3] of the increased risk conferred by sarcopenic obesity, defined as the co-presence of increased fat mass and decreased muscle mass on anthropometric measurements. Patients with sarcopenic osteoarthritis were weaker, thinner, malnourished, and had limited levels of physical activity.

Anthropometric measurements are not exclusive measures of adiposity and fat but also reflect the composite of muscle and bone mass. The state of the muscle mass both from a quantitative and qualitative point of view is of considerable importance in the pathogenesis of KOA, since the knee joint is mainly responsible for daily loading activities, which require the integrity of the knee-stabilizing muscles for load or vibration absorption [15].

Comparing pain and function in the three groups of patients stratified by BMI, the analysis revealed a direct proportionality relationship between BMI, pain, and functional impairment. As reported in Table 2, the group of normal-weight patients (BMI 18.5–24.9) showed lower VAS and GLFS scores compared to overweight (BMI 25–29.9) and obese (BMI 30–60) patients.

Indeed, regarding the stratification of patients according to the Kellgren–Lawrence Scale, it was observed that pain and locomotor abilities in walking and activities of daily living had higher scores in overweight patients and those with radiographically more severe knee osteoarthritis (K-L III).

In summary, our data indicate that a higher BMI is significantly associated with greater pain and increased difficulty in walking. Additionally, more severe arthritis is associated with greater difficulty in walking and a higher BMI.

Rehabilitation, which includes manual techniques and instrumental physical therapies, is considered a cornerstone of KOA management, and in the literature, several rehabilitative interventions have been described as effective for reducing pain and improving function in individuals with knee OA [16,17,18,19,20,21]. Exercise therapy is beneficial to patients with KOA for relieving pain, alleviating stiffness, improving function, and improving quality of life, representing a useful therapeutic option, especially for early knee osteoarthritis [17].

Comparing data related to pain and functional impairment in the two groups of patients divided by whether they underwent rehabilitative treatments or not, it emerged that the mean VAS was comparable between the two groups, while the functional impairment was generally greater in the group that performed rehabilitative treatments, as shown in Table 2. An important gap in our database is that physicians participating in the survey did not provide information regarding the specific rehabilitation therapies prescribed to their patients at the time of observation; therefore, we are not able to discuss which therapeutic intervention was more or less effective in the management of symptomatic knee osteoarthritis.

Regarding the pharmacological treatment of knee osteoarthritis, our data analysis reflects the trend reported in the recent literature that the most widely used KOA treatment in real-world settings is still non-steroidal anti-inflammatory drugs therapy, despite the evidence for serious adverse effects [22]. In our study, before the observation during the symptomatic phase, most patients predominantly used NSAIDs, secondly SYSADOAs, and a smaller portion paracetamol, corticosteroids, and opioids. The greater consumption of NSAIDSs compared to SYSADOAs before observation, which is not in keeping with the ESCEO guidelines that strongly recommended long-term background therapy with crystal-line glucosamine sulfate, is homogeneous in all subgroups of the sample divided based on BMI (Figure 2), K-L (Figure 3), and GLFS (Figure 4). However, after the observation, a notable decrease in NSAID prescription and an increase in SYSADOA utilization were also observed in all sample subgroups (Figure 2, Figure 3 and Figure 4), demonstrating the wide popularity gained by nutraceuticals as alternative treatments for KOA, whose advantage is that they are not associated with any side effects, although their clinical benefits have not been clearly established [23]. Indeed, in some papers, SYSADOAs were reported to be ineffective or showing arguably clinically unimportant treatment effects. Conversely, some studies have reported that glucosamine or condroithine are able to relieve KOA symptoms [24,25], and this evidence could probably explain our observation regarding obese patients, who presented a significant reduction in NSAIDs and an increase in SYSADOAs after the visit. Instead, there was no reduction in paracetamol intake, which was observed in non-obese patients.

An effective and widespread alternative with limited side effects in managing knee osteoarthritis is the administration of viscoelastic and/or anti-inflammatory substances through intra-articular injection.

Despite the fact that regenerative therapies are evolving nowadays, with the employment of innovative intra-articular formulations such as platelet-rich plasma, ozone gas, and mesenchymal stem cells, hyaluronic acid and corticosteroids still play an anti-inflammatory role in KOA and relieve pain and inflammation [26]. Indeed, in our study sample, a percentage rise in the performance of intra-articular knee injections was recorded after observation in all the four subgroups of patients divided by BMI, K-L, GLFS, and rehabilitation performance, as shown in Table 3. And this increase in the use of intra-articular injections is in line with the rules of common clinical practice, as well as with international guidelines, that if NSAIDs are contraindicated or ineffective [11], or for patients with contraindications for surgery or for those who have a negative response to other therapeutic approaches [27], intra-articular injections of hyaluronic acid and corticosteroids may be considered as an alternative therapeutic strategy. Therefore, based on the survey results, currently, intra-articular knee injections constitute a therapeutic option that is taken into account for the real-word management of knee osteoarthritis by general practitioners and specialists, particularly in severe cases or in those who did not respond to oral medication, although from the database, it was not possible to discriminate which type of cortisone and hyaluronic acid was chosen for intra-articular use.

## 5. Conclusions

The goals of treatment for symptomatic knee osteoarthritis are to relieve symptoms and slow cartilage degeneration, with a consequent improvement in quality of life and healthcare cost reduction.

Despite the limitations of this work, which lie in the incompleteness of the information that was extractable from the database, such as the inability to determine the specific type of rehabilitation undergone by the patient, lack of information on the specific protocols used during treatment, absence of details regarding post-treatment follow-up, lack of specifics regarding the number of intra-articular administrations, and the type of medications used for the intra-articular knee injections, we believe that this real-word analysis could be useful in recognizing the gap between scientific evidence and clinical practice.

Currently, there are no guidelines for standardized protocols using effective combinations of therapeutic exercises, physical agents, and medications to control the progression of knee osteoarthritis. We hope that our work can serve as a stimulus for new studies aimed at defining integrated therapeutic protocols for the optimal management of knee osteoarthritis in real-life outpatient settings.

## Figures and Tables

**Table 1:** Demographic and anthropometric characteristics of the sample.

	Total	Men	Women
Sex, number (%)	2656	1155 (45.2%)	1501 (54.8%)
Mean age (mean ± standard deviation)	67.2 ± 11.7	66.8 ± 12.5	67.6 ± 11
Weight (kg) (mean ± standard deviation)	74 ± 11.6	79.4 ± 9.7	69.9 ± 11.2
Mean height (cm) (mean ± standard deviation)	167.7 ± 8.3	173.7 ± 6.8	163.1 ± 6.2
Body Mass Index (BMI) (mean ± standard deviation)	26.4 ± 3.8	26.4 ± 3.1	26.4 ± 4.2

**Table 2:** VAS and GLFS scores in relation to the sample stratifications for BMI, K-L, and rehabilitation.

	VAS Mean and SD	GLFS Mean and SD	BMI Mean and SD
BMI (range)			
18.5–24.9 n: 891	5.9 ± 1.5 *	7.1 ± 4.2 ^	-
25–29.9 n: 1135	6.5 ± 1.5 *	8.2 ± 4.3 ^	-
30–60 n: 409	6.3 ± 1.6 **	10.2 ± 4.7 ^	-
K-L II n: 882	6.2 ± 1.5 ^^	7.6 ± 3.9 ***	26.7 ± 4.2 ^^^
K-L III n: 632	6.4 ± 1.5 ^^	11.3 ± 4.4 ***	28.7 ± 4.3 ^^^
Rehabilitation: YES n: 1389	6.1 ± 1.5	8.8 ± 4.4 °	
Rehabilitation: NO n: 1252	6.2 ± 1.6	7.6 ±4.3 °	

BMI, Body Mass Index; GLFS, Geriatric Locomotive Function Scale; K-L, Kellgren–Lawrence; VAS, Visual Analogic Scale. * *p* level 0.0465, ** *p* level 0.0006, one-way ANOVA with Tukey’s multiple comparison test. ^ *p* level 0.0001, one-way ANOVA with Tukey’s multiple comparisons test. ^^ *p* level 0.0115, unpaired *t* test. *** *p* level 0.0001, unpaired *t* test. ^^^ *p* level 0.0001, unpaired *t* test. ° *p* level 0.0001, unpaired *t* test.

**Table 3:** Intra-articular knee injection prior to and after observation in relation to the sample stratification for BMI, K-L, and rehabilitation.

	Intra-Articular Knee Injection	
	Before Observation %	After Observation %
BMI (range)		
18.5–24.9 n: 891	0.3%	6.8%
25–29.9 n: 1135	0.6%	6.6%
30–60 n: 409	0.5%	6.4%
K-L II n: 882	0.2%	8.6%
K-L III n: 632	0.5%	8.7%
GLFS < 6 n: 828	0.0%	6.4%
GLFS > 6 n: 1797	0.5%	7.9%
Rehabilitation: YES n: 1389	0.5%	8.9%
Rehabilitation: NO n: 1252	0.3%	5.6%

BMI, Body Mass Index; GLFS, Geriatric Locomotive Function Scale; K-L, Kellgren–Lawrence; VAS, Visual Analogic Scale.

## Data Availability

The data generated and analyzed during this study are included in this published article and are available from the corresponding author.

## Abbreviations

KOA, knee osteoarthritis; NSAIDs, non-steroidal anti-inflammatory drugs; ESCEO, European Society for Clinical and Economic Aspects of Osteoporosis, Osteoarthritis and Musculoskeletal Diseases; SYSADOAs, Symptomatic Slow-Acting Drugs for Osteoarthritis; BMI, Body Mass Index; K-L, Kellgren–Lawrence; VAS, Visual Analogic Scale; GLFS, Geriatric Locomotive Function Scale.
